# Comparison of ICD code-based diagnosis of obesity with measured obesity in children and the implications for health care cost estimates

**DOI:** 10.1186/1471-2288-11-173

**Published:** 2011-12-21

**Authors:** Stefan Kuhle, Sara FL Kirk, Arto Ohinmaa, Paul J Veugelers

**Affiliations:** 1 School of Public Health, University of Alberta, 650 University Terrace, Edmonton, AB, T6G 2T4, Canada; 2 School of Health Administration, Dalhousie University, Halifax, NS, Canada

## Abstract

**Background:**

Administrative health databases are a valuable research tool to assess health care utilization at the population level. However, their use in obesity research limited due to the lack of data on body weight. A potential workaround is to use the ICD code of obesity to identify obese individuals. The objective of the current study was to investigate the sensitivity and specificity of an ICD code-based diagnosis of obesity from administrative health data relative to the gold standard measured BMI.

**Methods:**

Linkage of a population-based survey with anthropometric measures in elementary school children in 2003 with longitudinal administrative health data (physician visits and hospital discharges 1992-2006) from the Canadian province of Nova Scotia. Measured obesity was defined based on the CDC cut-offs applied to the measured BMI. An ICD code-based diagnosis obesity was defined as one or more ICD-9 (278) or ICD-10 code (E66-E68) of obesity from a physician visit or a hospital stay. Sensitivity and specificity were calculated and health care cost estimates based on measured obesity and ICD-based obesity were compared.

**Results:**

The sensitivity of an ICD code-based obesity diagnosis was 7.4% using ICD codes between 2002 and 2004. Those correctly identified had a higher BMI and had higher health care utilization and costs.

**Conclusions:**

An ICD diagnosis of obesity in Canadian administrative health data grossly underestimates the true prevalence of childhood obesity and overestimates the health care cost differential between obese and non-obese children.

## Background

The prevalence of childhood overweight and obesity has reached epidemic proportions in Western countries [1]. One of the emerging and costly consequences of this epidemic is the increase in health care utilization by obese children for a number of conditions [2-4]. Documenting these disparities in health care use can help to better target prevention efforts and resource allocation. Administrative databases provide an invaluable tool in this respect as they capture information on physician-diagnosed conditions for a large sample of the population. However, a significant shortcoming of these databases for their use in obesity research is the lack of data on body weight. Previous studies have often linked utilization data with anthropometric data from surveillance systems, hospital charts, or surveys to identify overweight and obese children [2-5]. Such linkage may not always be feasible and other investigators have used an ICD-9/10 diagnosis of obesity from the administrative database to identify obese children [6-10]. Woo et al. [11] using data from a tertiary care hospital in the US showed that using an ICD-9 code-based diagnosis underestimated the true prevalence of obesity in children. However, children in a tertiary care hospital represent a rather selected population and any weight problem in these children may have been considered minor compared to the health problem that led to the admission. A weight problem is more likely to come to the attention of a pediatrician or general practitioner who commonly has a closer relationship with the family and sees most children on a regular basis. Hence, the former findings may not apply to population-based administrative databases that include data on physician visits and diagnoses such as Canadian provincial administrative health databases. Canada's publicly funded universal single provider health care system poses no financial barriers to seeking health services and tracks use and costs of hospital and physician services in each province. The objective of the current paper was twofold: i) to investigate the sensitivity and specificity of an ICD code-based diagnosis of obesity from administrative health data for identifying measured obesity in Canadian children; and ii) to compare health care costs in obese children identified by either method.

## Methods

The data used in the present study come from a linkage of a population-based survey in elementary school children with administrative health data.

### Children's Lifestyle and School Performance Study

The CLASS (Children's Lifestyle and School Performance Study) is a population-based survey of Grade 5 students and their parents in the Canadian province of Nova Scotia in 2003 [12]. The study consisted of a questionnaire that was completed at home by the parents; a student questionnaire and a Canadianized version of the Harvard Youth/Adolescent Food Frequency Questionnaire administered to the students in the schools by study assistants; and a single measurement of the students' standing height and body weight. Standing height was measured to the nearest 0.1 cm after students had removed their shoes; body weight was measured in light indoor clothing without shoes to the nearest 0.1 kg on calibrated digital scales. In addition to the above information, participating parents were asked to provide their Nova Scotia Health Insurance number and informed consent to allow future linkage with birth and administrative health databases.

Of the 291 public schools in Nova Scotia (> 97% of students attend public schools) with grade 5 classes, 282 (96.9%) participated in the study. The average rate of return of questionnaire and consent form was 51.1% per school. One of the seven provincial school boards did not allow anthropometric measurements. A total of 4298 students participated in the study and had their height and weight measured.

### Administrative health data

The CLASS data were linked with Nova Scotia administrative health data consisting of the Medical Services Insurance (MSI) database, and the Canadian Institute for Health Information Discharge Abstract Database (CIHI DAD). ICD diagnosis data were available from 1992 (i.e. the child's year of birth) up to 2006. The MSI database is administered by Medavie Blue Cross for the province of Nova Scotia and contains administrative records for each insured health service rendered by a physician (including emergency room visits) and paid for by the Nova Scotia provincial healthcare system. The CIHI DAD contains a comprehensive administrative transcription of each admission to a Nova Scotia hospital facility. Both of these databases contain individual patient-level information including patient demography (age, gender, location, etc), attending physicians, diagnoses and procedures performed, service transfers while in hospital, specialty services received (e.g. physiotherapy, occupational therapy), and case complexity (e.g. resource intensity weight). Aggregate costs of health care episodes for physician visits and hospitalizations were obtained from the Ontario Case Costing Initiative [13]. Costs were adjusted to 2006 Canadian Dollars using the Canadian Consumer Price Index [14]. A combination of deterministic and probabilistic matching was used to link the administrative health datasets with the CLASS study data. Of the 4412 students in the CLASS study with valid home survey and school information, 4380 could be linked with information in the administrative datasets. In the remaining children, parents had provided an invalid or no health insurance number. A total of 3399 out of the 4380 children had a measured BMI.

### Obesity Definitions

Measured obesity was defined using the body mass index (BMI) cut-off points based on the CDC growth charts [15], which were used by physicians in Canada until 2010 to identify overweight and obese children. At the time of BMI measurement, children were 10 to 11 years old. To enable comparability with an ICD code-based obesity diagnosis, which does not have an 'overweight' category, overweight children were considered 'normal weight' for the purposes of this analysis. An ICD code-based diagnosis of obesity was made when the child had one or more ICD-9 code of 278 or one or more ICD-10 code of E66-E68 as a primary or secondary diagnosis from either a physician visit or a hospital stay.

### Statistical Analysis

Point estimates and 95% confidence intervals for sensitivity, specificity, and Cohen's Kappa were calculated for ICD code-based obesity diagnosis (from administrative health data between 2002 and 2004) vs. the gold standard obesity diagnosis based on measured BMI (in 2003; at age 10/11 years). To assess the effect of misclassification resulting from using an ICD code-based diagnosis of obesity, the association between obesity and total health care costs incurred between 2003 to 2006 was modeled using regression models based on measured and ICD code-based obesity, respectively. Cost differentials between obese and normal weight children were expressed as "cost ratios" by modeling the log costs and then exponentiating the regression coefficients. Details on the cost model methodology have been published elsewhere [5]. The BMI and the number of physician visits of obese (as per measured BMI) children with an ICD code of obesity was compared to obese children with no ICD code of obesity using a t-test. All analyses were done in 3230 children with at least one health care provider contact between 2002 and 2004.

This study, including data collection, parental informed consent forms, and data linkage was approved by the Health Sciences Human Research Ethics Board of Dalhousie University, the IWK Health Centre Research Ethics Board, the Reproductive Care Program Joint Data Access Committee and the Dalhousie University Population Health Research Unit Data Access Committee.

## Results

The prevalence of measured overweight (excluding obesity) and obesity in 2003 in the sample based on the CDC BMI cut-offs [15] were 18.0 and 16.4%, respectively.

Forty-seven out of 3230 children (1.5%) who had at least one recorded health care provider contact between 2002 and 2004 had an ICD diagnosis for obesity during that period. In the majority of cases (77%), the diagnosis of obesity was made by a general practitioner, while the remaining diagnoses came from a pediatrician. Seventy percent of children received only one diagnosis of obesity between 2002 and 2004, approximately 11% had two diagnoses, and 19% had 3 or more obesity diagnosis codes. More than 90 percent of ICD diagnoses of obesity were primary diagnoses.

The sensitivity of an ICD diagnosis of obesity between 2002 and 2004 for identifying a child with measured obesity in 2003 was 7.4% (95%CI 5.3; 9.9), while specificity was 99.7% (95%CI 99.4; 99.9). Agreement between ICD diagnosis and measured obesity was poor at 0.11 (95%CI 9.4; 12.9).

Modeling the association between obesity and total health care costs using different definitions of obesity showed that an ICD code-based diagnosis of obesity (2002-2004) compared to measured obesity (2003) overestimated the true cost differential by a wide margin (2.08 [95%CI 1.47; 2.93] vs. 1.16 [95%CI 1.01; 1.32]).

Children who were correctly diagnosed as obese in the administrative health data between 2002 and 2004 differed from those who were obese but not diagnosed through an ICD code in that they had a significantly larger BMI (29.9 vs. 27.0 kg/m^2^, p < 0.0001). Twenty-five percent of obese children who did not have an ICD code of obesity had a BMI of 28.5 kg/m^2 ^or higher (up to a maximum of 44.0 kg/m^2^). The average number of physician visits between 2002 and 2004 in those correctly identified as obese was significantly higher than in those who were obese but not diagnosed (15.5 vs. 9.7, p = 0.0003).

## Discussion

This study examined for the first time how well an ICD code of obesity identifies obese children in Canadian administrative health data. The sensitivity of an ICD code-based obesity diagnosis for detecting measured obesity was low, only 7% of obese children were correctly identified. Those correctly identified had higher health care utilization than those without an ICD diagnosis of obesity.

An ICD diagnosis of obesity has been used previously to identify obese children in administrative databases [6-10]. A recent study showed that the majority of children (> 90%) with measured obesity did not receive an ICD diagnosis of obesity during their inpatient stay at a tertiary care hospital in Ohio [11]. We had speculated that the sensitivity of an ICD diagnosis might be higher if both physician visits and hospital stays are used since children see their general practitioner or pediatrician far more often than being admitted to a hospital, and a weight problem may be more likely to be picked up during a well child visit or a consultation for a minor ailment. This hypothesis is supported by the fact that all children in our sample received the ICD diagnosis of obesity during a physician visit (either general practicioner or pediatrician).

The consequence of the poor sensitivity of the ICD code for obesity is that administrative data will grossly underestimate the true population prevalence of obesity. We were also interested in examining whether this misclassification is differential with regard to health care utilization. When comparing the costs in obese children and normal weight children, costs were 16% higher in children with measured obesity. This cost differential increased to 108% when the analysis was based on an ICD diagnosis of obesity between 2002 and 2004. That is, using an ICD diagnosis compare health care utilization between obese and non-obese children severely overestimated health care costs for obese children. A possible explanation is that physicians are more likely to diagnose obesity in a child if multiple, potentially obesity-related, co-morbidities are present. This argument is further supported by the finding that the number of physician visits was significantly higher in those correctly identified as obese. An alternative explanation could be that with the identification of obesity, physicians scheduled more frequent evaluations of the patients. However, a comparison of the number of physician visits three years before and after the ICD diagnosis showed no difference (p = 0.91, data not shown). Hampl et al. [4] examining inpatient utilization in a pediatric primary care centre in the US reported higher health care costs for children with diagnosed obesity compared to those with undiagnosed obesity. By contrast, Woo et al. [11] found that children with diagnosed obesity had shorter hospital stays and fewer hospital discharges than both non-obese and undiagnosed obese patients. The apparent discrepancy may be explained by the fact that the study was done in a tertiary care hospital while some health conditions that are more common in obese children are primarily treated on an outpatient basis (e.g. asthma, type 2 diabetes). Children with diagnosed obesity in Woo's study were more likely to have primary diagnoses of mental health, endocrine, and musculo-skeletal disorders compared to children with undiagnosed obesity. This finding may indicate that the presence of a 'typical' obesity-related disorder increases the likelihood of receiving a diagnosis of obesity [11].

Besides the methodological aspects, our findings raise some concern about identification of obesity in the primary care system. General practitioners and pediatricians have a critical role in the diagnosis, education and management of overweight and obesity as they constitute the first point of contact within the health care system. An obese child that is not diagnosed (and not counseled) is a lost opportunity for secondary prevention. As shown in our analysis, less than 10% of obese children are diagnosed as obese by an ICD code. Even more concerning is that a quarter of children with undiagnosed obesity had a BMI between 29 and 44 kg/m^2^, which is well beyond the age-specific obesity cut-off of approximately 23 kg/m^2 ^[15] and clearly associated with health risks. The marked discrepancy between the ICD-based prevalence between 2002 and 2004 (1.5%) and that of measured obesity (16.4%) suggests that obesity was frequently overlooked or the issue was avoided by the physicians. On the other hand, ICD codes in administrative health data are primarily collected for billing purposes and the lack of an ICD code of obesity for an obese child may not necessarily indicate that a physician did not address the problem in the consultation. One may also argue that the Canadian health care system does not have the capacity to manage childhood overweight and obesity, and that the problem is best tackled by primary prevention measures.

The strengths of the current paper are the use of longitudinal administrative data from a universal single provider health care system linked with a population-based survey, and the coverage of both physician visits and hospital stays. Our findings are limited by the single BMI measurement at age 10/11 years and the lack of synchronicity between the BMI measurement and the physician visit/hospital stay. However, obesity is the result of long-term lifestyle habits and not expected to change within a relatively short time frame. Another limitation is the response rate of 51%, which may have resulted in a selected sample. If the non-responders in the survey had a different probability of being identified as obese by their physicians compared to the children participating in the survey, the results would be biased. On the other hand, sensitivity and specificity are not affected by the prevalence of a condition, and therefore a higher or lower prevalence of obesity in the non-responders would not influence the results. The non-responders would also be likely to be seen by the same physicians as their participating peers and hence the sensitivity and specificity can be expected to be comparable between the two groups.

## Conclusions

An ICD diagnosis of obesity grossly underestimates the true prevalence of childhood obesity in Canadian administrative health data. Using the ICD code for obesity to identify obese children will overestimate the cost differential between obese and non-obese children.

## Competing interests

The authors declare that they have no competing interests.

## Authors' contributions

SK conceived the study, analyzed the data and wrote the manuscript. SFLK acquired the data and critically revised the manuscript. AO conceived the study and critically revised the manuscript. PJV acquired the data, interpreted the data, and critically revised the manuscript. All authors read and approved the final manuscript.

## Pre-publication history

The pre-publication history for this paper can be accessed here:

http://www.biomedcentral.com/1471-2288/11/173/prepub

## References

[B1] Janssen I Katzmarzyk PT Boyce WF Vereecken C Mulvihill C Roberts C Currie C Pickett W Comparison of overweight and obesity prevalence in school-aged youth from 34 countries and their relationships with physical activity and dietary patterns Obes Rev 2005 6 123 132 10.1111/j.1467-789X.2005.00176.x 15836463

[B2] Estabrooks PA Shetterly S The prevalence and health care use of overweight children in an integrated health care system Arch Pediatr Adolesc Med 2007 161 222 227 10.1001/archpedi.161.3.222 17339502

[B3] Buescher PA Whitmire JT Plescia M Relationship between body mass index and medical care expenditures for North Carolina adolescents enrolled in Medicaid in 2004 Prev Chronic Dis 2008 5 A04 18081993 PMC2248788

[B4] Hampl SE Carroll CA Simon SD Sharma V Resource utilization and expenditures for overweight and obese children Arch Pediatr Adolesc Med 2007 161 11 14 10.1001/archpedi.161.1.11 17199061

[B5] Kuhle S Kirk S Ohinmaa A Yasui Y Allen AC Veugelers PJ Use and cost of health services among overweight and obese Canadian children Int J Pediatr Obes 2010 10.3109/17477166.2010.486834 20874077

[B6] Wang G Dietz WH Economic burden of obesity in youths aged 6 to 17 years: 1979-1999 Pediatrics 2002 109 E81 10.1542/peds.109.5.e81 11986487

[B7] Marder WD Chang S Childhood Obesity: Costs, Treatment Patterns, Disparities in Care and Prevalent Medical Conditions http://www.medstat.com/pdfs/childhood_obesity.pdf

[B8] Vellinga A O'Donovan D De La Harpe D Length of stay and associated costs of obesity related hospital admissions in Ireland BMC Health Serv Res 2008 8 88 10.1186/1472-6963-8-88 18426608 PMC2377242

[B9] Woolford SJ Gebremariam A Clark SJ Davis MM Incremental hospital charges associated with obesity as a secondary diagnosis in children Obesity (Silver Spring) 2007 15 1895 1901 10.1038/oby.2007.224 17636109

[B10] Woolford SJ Gebremariam A Clark SJ Davis MM Persistent gap of incremental charges for obesity as a secondary diagnosis in common pediatric hospitalizations J Hosp Med 2009 4 149 156 10.1002/jhm.388 19301381

[B11] Woo JG Zeller MH Wilson K Inge T Obesity Identified by Discharge ICD-9 Codes Underestimates the True Prevalence of Obesity in Hospitalized Children J Pediatr 2009 154 327 331 10.1016/j.jpeds.2008.09.022 18950792 PMC4664085

[B12] Veugelers PJ Fitzgerald AL Prevalence of and risk factors for childhood overweight and obesity Can Med Assoc J 2005 173 607 613 10.1503/cmaj.050445 PMC1197160 16157724

[B13] OCCI Ontario Case Costing Initiative http://www.occp.com/

[B14] Statistics Canada Consumer Price Index, historical summary http://www40.statcan.gc.ca/l01/cst01/econ46a-eng.htm

[B15] Kuczmarski RJ Ogden CL Grummer-Strawn LM Flegal KM Guo SS Wei R Mei Z Curtin LR Roche AF Johnson CL CDC growth charts: United States Adv Data 2000 1 27 11183293

